# A seven-gene prognostic signature predicts overall survival of patients with lung adenocarcinoma (LUAD)

**DOI:** 10.1186/s12935-021-01975-z

**Published:** 2021-06-06

**Authors:** Aisha Al-Dherasi, Qi-Tian Huang, Yuwei Liao, Sultan Al-Mosaib, Rulin Hua, Yichen Wang, Ying Yu, Yu Zhang, Xuehong Zhang, Chao Huang, Haithm Mousa, Dongcen Ge, Sufiyan Sufiyan, Wanting Bai, Ruimei Liu, Yanyan Shao, Yulong Li, Jingkai Zhang, Leming Shi, Dekang Lv, Zhiguang Li, Quentin Liu

**Affiliations:** 1 Center of Genome and Personalized Medicine, Institute of Cancer Stem Cell, Dalian Medical University, Dalian, 116044 Liaoning People’s Republic of China; 2 Department of Biochemistry, Faculty of Science, Ibb University, Ibb, Yemen; 3 Yangjiang Key Laboratory of Respiratory Diseases, Yangjiang People’s Hospital, Yangjiang, Guangdong Province People’s Republic of China; 4 Department of Computer Science and Technology, Sahyadri Science College, Kuvempu University, Shimoga, Karnataka India; 5 State Key Laboratory of Genetic Engineering, School of Life Sciences and Human Phenome Institute, Fudan University, 2005 Songhu Road, Shanghai, 200438 People’s Republic of China; 6 Department of Clinical Biochemistry, College of Laboratory Diagnostic Medicine, Dalian Medical University, Dalian, 116044 Liaoning People’s Republic of China

**Keywords:** Lung adenocarcinoma (LUAD), Overall survival, Risk score, Prognostic signature

## Abstract

**Background:**

Lung adenocarcinoma (LUAD) is one of the most common types in the world with a high mortality rate. Despite advances in treatment strategies, the overall survival (OS) remains short. Our study aims to establish a reliable prognostic signature closely related to the survival of LUAD patients that can better predict prognosis and possibly help with individual monitoring of LUAD patients.

**Methods:**

Raw RNA-sequencing data were obtained from Fudan University and used as a training group. Differentially expressed genes (DEGs) for the training group were screened. The univariate, least absolute shrinkage and selection operator (LASSO), and multivariate cox regression analysis were conducted to identify the candidate prognostic genes and construct the risk score model. Kaplan–Meier analysis, time-dependent receiver operating characteristic (ROC) curve were used to evaluate the prognostic power and performance of the signature. Moreover, The Cancer Genome Atlas (TCGA-LUAD) dataset was further used to validate the predictive ability of prognostic signature.

**Results:**

A prognostic signature consisting of seven prognostic-related genes was constructed using the training group. The 7-gene prognostic signature significantly grouped patients in high and low-risk groups in terms of overall survival in the training cohort [hazard ratio, HR = 8.94, 95% confidence interval (95% CI)] [2.041–39.2]; P = 0.0004), and in the validation cohort (HR = 2.41, 95% CI [1.779–3.276]; P < 0.0001). Cox regression analysis (univariate and multivariate) demonstrated that the seven-gene signature is an independent prognostic biomarker for predicting the survival of LUAD patients. ROC curves revealed that the 7-gene prognostic signature achieved a good performance in training and validation groups (AUC = 0.91, AUC = 0.7 respectively) in predicting OS for LUAD patients. Furthermore, the stratified analysis of the signature showed another classification to predict the prognosis.

**Conclusion:**

Our study suggested a new and reliable prognostic signature that has a significant implication in predicting overall survival for LUAD patients and may help with early diagnosis and making effective clinical decisions regarding potential individual treatment.

**Supplementary Information:**

The online version contains supplementary material available at 10.1186/s12935-021-01975-z.

## Background

Despite the advancements in lung cancer treatment, non-small lung cancer (NSCLC) remains one of the most common types and the leading cause of cancer-associated mortality among men and women worldwide [1]. NSCLC and small cell lung cancer (SCLC) are the two major types of lung cancer. The two main types of NSCLC are lung squamous cell carcinoma (LUSC) and lung adenocarcinoma (LUAD) [2]; thus, these histological subtypes may determine the choice of treatment [2, 3]. The poor prognosis and short survival of lung cancer patients may be associated with the development of pulmonary hypertension (PH) due to blockage of tumor cells in the pulmonary vessels [4, 5]. In the last few years, the absolute and relative frequencies of lung cancer’s incidence and mortality have risen dramatically worldwide [6, 7]. Overall, the 5-year survival rate for lung cancer is 19% [8]. A total of 235,760 new cases of lung cancer and 131,880 deaths from lung cancer were expected to occur in 2021 [9].

Lung adenocarcinoma (LUAD) is one of the main subtypes of lung cancer [2]. However, most of patients with lung adenocarcinoma are diagnosed in the late stages or in the metastatic stage (third or fourth stage) of the disease; significant and longer survival rates can be achieved for those who are diagnosed at an early stage, but in advanced stages, curative treatment options are prolonged and limited, resulting in poor prognosis and low survival rates [10]. Time is the crucial factor for all patients with cancer; in addition to the fact that lung adenocarcinoma (LUAD) is a heterogeneous group of diseases and individual differences of patients at the same pathological stages that may cause distinct prognoses for each patient, all these reasons have led to emergence of a clearly unmet medical need for identifying the accurate and promising prognostic biomarker and efficient therapeutic targets that can aid the clinicians by facilitating the accurate and early diagnosis of lung adenocarcinoma, enhancing poor survival of LUAD patients and guiding customized treatment [11, 12].

Recently, various studies have been conducted to identify a lot of biomarkers related to prognosis, drug resistance and diagnosis to guide long-term prognosis in patients with NSCLC. Nevertheless, many studies have been limited to a single biomarker such as a SLC2A1 and PKM [13, 14] or a small set of samples, causing inaccuracies and unavailability of biomarkers. Therefore, the biomarker found through the study of high-throughput gene expression profiles and built through a combination of multiple biomarkers is more promising [15]. In addition, clinical variables and pathohistological characteristics of the tumor have been used as biomarkers to predict patient’s overall survival. The most commonly used parameter to assess the prognosis and mentor the treatment of patients with cancer is the TNM classification system [16]. However, predicting the survival of patients with lung adenocarcinoma (LUAD) by a single parameter or a single gene is one of the difficulties that lead to distinct prognoses for each patient due to the effect of genetic heterogeneity of the LUAD and the wide variations in patient’s outcomes [11, 12, 17]. Therefore, several studies began to identify gene biomarkers related to LUAD prognosis [18–20]. Prognostic gene signature based on combination of multiple genes plays an important role in guiding and assisting clinicians in choosing the appropriate treatment method, highlighting about the cancer progression as well as detecting possible new treatment targets. Thus it is important to establish an expression-based gene signature to predict the outcomes and progress of LUAD patients.

In the current study, we conducted univariate cox proportional hazard regression analysis, lasso regression and multivariate cox proportional hazard regression analysis to screen new prognostic-related genes and establish a prognostic signature as a biomarker using LUAD data from Fudan University. ROC curve and kaplan–Meier analysis were used to evaluate the prognostic performance of the signature. Then prognosis value of the signature was further validated using a LUAD dataset from TCGA database. Furthermore, we performed stratification analysis to estimate the performance of the signature in different subgroups, beyond that, we investigated the possible biological functions of the key genes in the signature. Overall, our study suggested that the 7-gene signature has successfully and effectively contributed to predicting survival for LUDA patients, and these genes may become a new target for future treatment.

## Materials and methods

### Data source

The raw data of RNA-sequencing (RNA.seq) and relevant clinical information (including survival information) of 102 patients with LUAD were obtained from Fudan university as the training group. For the validation group, the data related to gene expression and clinical information of lung adenocarcinoma (LUAD-TCGA) were downloaded from the TCGA database (https://portal.gdc.cancer.gov/) and comprised a total of 594 (535 tumor sample and 59 normal samples) adenocarcinoma cases. Samples without sufficient clinical information were excluded from both the training and validation groups. The main characteristics of the analysis included the following: age, tumor size, sex, pT-stage, pathologic stage, and history of smoking; details of patient clinical information are described in Table 1. Approximately 48% of the samples were males, while 52% are females, and the participating age ranged from 37 to 84 years, with a median age of 61.5 years. Data were analyzed according to the ethical standards of the university review board (Fudan University Shanghai Cancer Center Institutional Review Board No. 090977-1). Collecting the samples from patients was conducted by the tissue bank of Fudan University Shanghai Cancer Center after the consent of patients or their relatives was obtained [21].

**Table 1:** The clinical information of patients with lung adenocarcinoma (LUAD), training (Fudan) and validation (TCGA) cohorts

Fudan (training group)	
Age—year (no.)	
< 60 year	40
≥ 60 year	62
Pathological_stage no	
IA	59
IB	27
IIIA	16
Smoking status—no	
Former/current	31
Never	71
Tumor_size—no	
< 3 cm	70
≥ 3 cm	32
Sex—no	
Male	49
Female	53
T—no	
T1a	40
T1b	27
T2a	33
T2b	1
T4	1
TCGA (validation group)	
Age—year (no.)	
< 60 year	87
≥ 60 year	240
Stage—no	
I	168
II	78
III	60
IV	21
N—no	
N0/N1	208/67
N2/N3	51/1
Sex—no	
Male	160
Female	167
T—no	
T1	100
T2	182
T3	28
T4	17
M—no	
M0	306
M1	21

### Determination of differentially expressed genes (DEGs) in LUAD

For generating the gene expression data in our study, the reads were mapped against the human genome (hg38) using STAR2 software [22]. The mapped reads with quality of more than 10 were selected using Samtools. The read counts per gene were defined using feature count [23] as the reference transcriptome. Differential expression analysis was performed using edgeR R package [24], and the tumor samples were compared to their matched normal samples to identify DEGs. The selected genes are significantly differentially expressed between tumor and normal samples and their FDR < 0.05 and absolute log2 fold change (logFC) > 1.

### Constructing a seven-gene prognostic signature

First, DEGs (n = 2725 PC) in the Fudan dataset were used to screen out the prognostic-related genes by using Kaplan–Meier (K-M) analysis. These screened genes were verified in 719 patients with lung adenocarcinoma (LUAD) from the Kaplan–Meier Plotter (http://kmplot.com/) [25–27]. To obtain the novel prognostic-related genes, preferably those that were not reported in lung cancer, we confirmed the reported genes and removed them to build a novel genetic signature. Second, for the non reported prognostic-related genes, univariate cox proportional hazard regression and LASSO regression analysis were used sequentially to evaluate the reliability of prognostic independent genes by using R packages, “survival” and “glment” respectively [28]. A P value of less than 0.05 was used as a cutoff to define and select the candidate genes related to patients’ survival. Finally, a multivariate cox proportional hazard regression analysis was performed to recognize the corresponding coefficients of LUAD prognostic signature by using “survminer” and “survival”R packages. We used the hazard ratio (HR) of each gene, to distinguish the protective genes from risk genes where the HR > 1 indicates that genes are risk genes and are protective genes otherwise (HR < 1).

The risk scoring for each patient was estimated using the (Eq. 1) to calculate the expression values pertaining to the selected genes weighted by regression coefficients in multivariate cox regression analysis.

1 $${\mathbf{Risk}}\;{\mathbf{Score = }}\sum\nolimits_{i - 1}^{n} {{\rm E}xp_{i} } * \mathop C\nolimits_{i}^{HR}$$

where n is the number of selected prognostic genes, E*xp*_i_ is the expression value of the prognostic gene i, and $C_{i}^{HR}$ is the estimated regression coefficient for the corresponding gene i in the multivariate cox regression analysis. Subsequently, the median prognostic score was used to differentiate between the high- and low-risk groups. The patients with lower risk than median value were assigned to the low-risk group, while the others were assigned to the high-risk group. Each of the K–M curve and the log-rank test was implemented using the “survival” R package to evaluate the survival analysis for each set. Then the prognostic performance of the prognostic score model was measured using the ROC curve by comparing the area under the respective receiver operating characteristic curve, and the “survivalROC” package was used in R to draw a ROC and then calculate the AUC.

### EGFR and KRAS mutation analysis

In order to identify patients with EGFR (Epidermal Growth Factor Receptor) and KRAS (Kirsten rat sarcoma viral oncogene homolog) mutations in the LUAD dataset, the whole exome sequencing (WES) data obtained from Fudan University was analyzed. Somatic mutations were filtered using Mutect2 under the following criteria: (i) the difference of mutant allele fraction (MAF) between the tumor and normal sample in the same patient was more than one percent; (ii), in both tumor and normal samples, the sequencing coverage was more than 200; (iii), the alternative readings in the tumor samples were more than10; (iv), the corrected p value was less than 0.05. SNVs were annotated using ANNOVAR, and further filtered with population frequency in ExAC, 1000 Genomes and dbSNP138. Then the correlation between EGFR and/or KRAS mutant patients and the gene expression of the seven prognostic genes was determined by using Wilcoxon test. Statistical significance was set as P < 0.05.

### Functional enrichment analysis

In order to explore the potential biological functions and pathways relationship in the seven prognostic genes, OmicsBean (http://www.omicsbean.cn/) online database was used. Using a functional annotation tool in omicsbeen, significantly enriched gene ontology (GO) terms and Kyoto Encyclopedia of Genes and Genomes (KEGG) pathways were achieved at threshold P value < 0.05. The annotations and background species for GO and KEGG pathways were set as Homo sapiens in omicsbeen.

### Statistical analysis

The K-M analysis was used to evaluate the differences in patients’ survival time between the high- and low-risk groups of patients with lung adenocarcinoma. The P values and HR (95% confidence interval) were determined by log-rank test and univariate cox regression analysis to detect the significant differences between the groups. Multivariate cox regression analysis and stratification analysis were performed to evaluate the independence of the risk score model. ROC curve was used to estimate the performance of gene prognostic signature by comparing the AUC. Statistical significance was identified as P ≤ 0.05. All statistical analyses were performed using version 3.5.1 of the R language.

## Results

### Patients characteristic

The median age of patients with LUAD during diagnosis was 61.5 years (ranging from 37 to 84 years). Adenocarcinoma was the histological subtype for all patients in the current study. In addition, 48% (n = 49) of our sample group were males and 52% were females (n = 53). Output status for all patients was either 0 or 1. Seventy-one patients (70%) have not smoked before, and 31 patients (30%) were former/current smokers. Fifty-nine patients (58%) had stage IA, 27 patients (26.4%) had stage IB, and 16 patients (15.6%) had stage IIIA (Table 1) (Additional file 1: Table S1). The patients have not received any neoadjuvant treatment.

### Identification of survival-related genes of lung adenocarcinoma (LUAD) patients

**Fig. 1:**
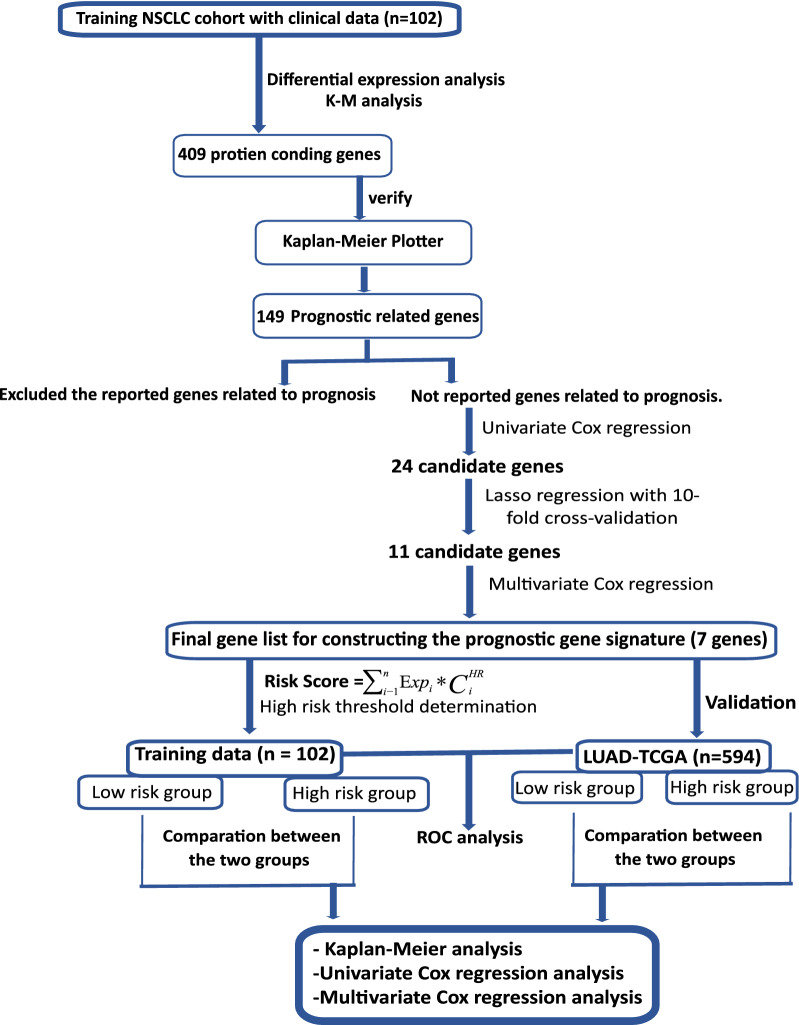
Flowchart of data processing, analysis, and validation in the current study

K–M analysis was used to establish the relationship between gene expression and the patient’s overall survival in the training cohort. We identified 409 protein-coding genes associated with overall survival, and these genes were verified by the Kaplan–Meier plotter database consisting 719 patients with lung adenocarcinoma. A total of 149 genes log-rank P value ≤ 0.05 were associated with LUAD survival. Of those, 31 genes have not been reported in patients with LUAD and used to conduct the next analyses to develop a prognostic signature model (Fig. 1). (Additional file 2: Table S2) shown the 31 unreported genes associated with LUAD survival.

### Construction of a 7-gene prognostic signature

**Fig. 2:**
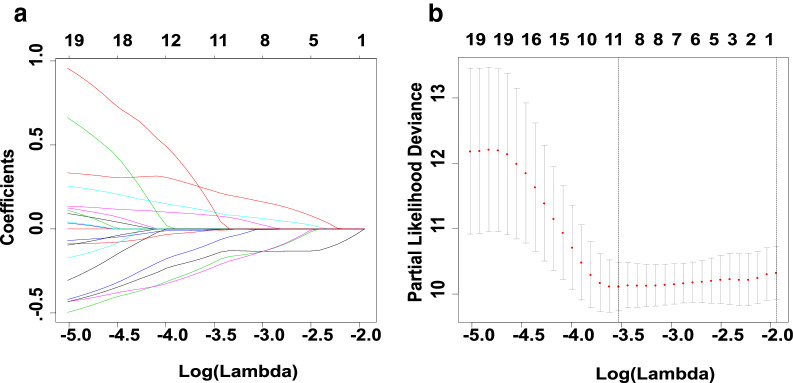
Identification and establishment of the seven-gene prognostic signature in patients with LUAD by LASSO regression model. **a** Genes are represented by the lines of various colors. The coef reach zero in some genes when the lambda value increases, and this indicates that those genes have no effect on the model. **b** The deviance of tenfold cross-validation obtained 11 prognostic genes.The best model depends on the minimum value of partial likelihood deviance

Survival-related genes that have not been reported in lung adenocarcinoma (n = 31 genes) from the training set were exposed for univariate cox regression analysis and LASSO regression analysis. Then, 24 genes were identified via the univariate regression analysis. LASSO regression analysis was performed to further identify the 24 genes that are significantly associated with the prognosis in patients with LUAD. Tenfold cross-validation was run to obtain the optimal λ value that came from the minimum partial likelihood deviance. The minimum value of the lambda for the optimal risk score model was 0.021940, as this value was associated with the 24 genes that were significantly correlated with the patient’s overall survival (Fig. 2). Multivariate cox proportional hazard regression analysis was performed on the 11 genes obtained from LASSO regression analysis. A total of seven genes were finally identified as the key genes in the prognostic model: UCN2, RIMS2, CAVIN2, GRIA1, PKHD1L1, PGM5, and CLIC6, which used for constructing the prognostic risk score for LUAD in the training group (Fig. 1). The seven gene-based risk score was constructed based on their coefficient of risk score model (Eq. 2):

2 $${\mathbf{Risk}} \, {\mathbf{score}} \, = \, ( - {\mathbf{0}}.{\mathbf{3658}}*{\mathbf{ExpGRIA1}}) \, + \, ({\mathbf{0}}.{\mathbf{5701}}*{\mathbf{ExpUCN2}}) \, + \, ( - {\mathbf{0}}.{\mathbf{601}}*{\mathbf{ExpPKHD1L1}}) \, + \, ({\mathbf{0}}.{\mathbf{2192}}*{\mathbf{ExpRIMS2}}) \, + \, \left( { - {\mathbf{0}}.{\mathbf{3617}} \, * \, {\mathbf{ExpPGM5}}} \right) \, + \, \left( { - {\mathbf{0}}.{\mathbf{6036}} \, * \, {\mathbf{ExpCLIC6}}} \right) \, + \, ({\mathbf{1}}.{\mathbf{1686}}* \, {\mathbf{ExpCAVIN2}}).$$

The information related to seven genes is shown in Table 2. Finally, a set of seven genes, including (n = 2) the risky gene (HR > 1) and (n = 5) the protective genes (HR < 1), was examined. Table 3 shows the prognostic correlation of seven genes with the survival of patients with LUAD in the training and validation groups.

**Table 2:** Overall information of the seven genes for constructing the prognostic signature

Gene ID	Gene symbol	Gene type	Chromosome	Gene start (bp)	Gene end (bp)
ENSG00000155511	GRIA1	Protein coding	chr5	153489615	153813869
ENSG00000145040	UCN2	Protein coding	chr3	48561727	48563773
ENSG00000205038	PKHD1L1	Protein coding	chr8	10936247	10953030
ENSG00000176406	RIMS2	Protein coding	chr8	10350078	10425604
ENSG00000154330	PGM5	Protein coding	chr9	68328308	68531061
ENSG00000159212	CLIC6	Protein coding	chr21	34669389	34718227
ENSG00000168497	CAVIN2	Protein coding	chr2	19183432	19184725

**Table 3:** Univariate cox regression analysis of seven-genes and OS of lung adenocarcinoma patients in both data

Genes	Training data		TCGA (Validation data)	
	HR (95% CI)	P	HR (95% CI)	P
GRIA1	0.7019 (0.5734–0.8593)	0.000604	0.87236 (0.817–0.9315)	4.46E−05
UCN2	1.4564 (1.139–1.863)	0.00276	1.12577 (1.049–1.208)	0.00102
PKHD1L1	0.7339 (0.5977–0.9011)	0.00314	0.86498 (0.7951–0.941)	0.000734
RIMS2	1.22874 (1.056–1.429)	0.00756	1.08175 (1.028–1.138)	0.00248
PGM5	0.6397 (0.4442–0.9212)	0.0163	0.88238 (0.8–0.9732)	0.0123
CLIC6	0.7442 (0.566–0.9785)	0.0344	0.87684 (0.8216–0.9358)	7.62E−05
CAVIN2	0.6865 (0.4711–1)	0.0503	0.91561 (0.8289–1.011)	0.0823

### The validation of 7-gene prognostic signature

**Fig. 3:**
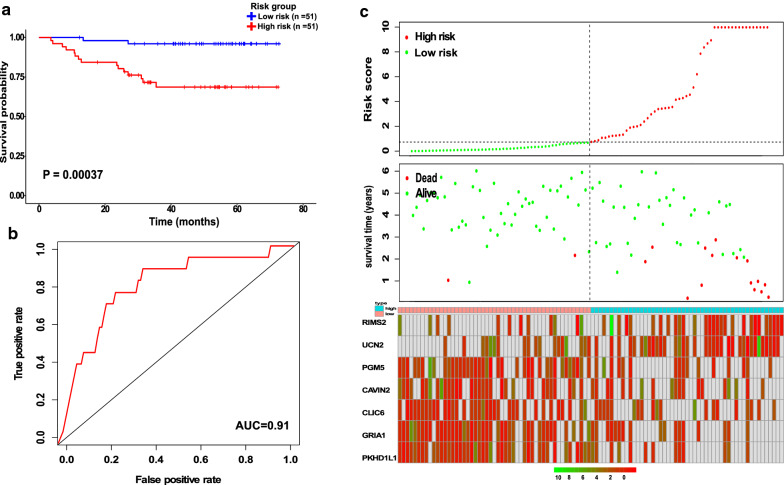
The prognostic performance of the 7-gene prognostic signature in the training group. **a** Kaplan–Meier survival analysis of the seven-gene prognostic signature. (**b** The receiver operating characteristic (ROC) curve analysis of seven-gene prognostic signature. **c** The distribution of risk scores (upper panel), the distribution of survival time (middle panel), and seven-gene expression profiles (bottom panel). Black dotted lines (median risk score) divide patients into low- and high-risk groups. Patients in the high-risk group are represented by red lines and dots. Patients in the low-risk group are represented by green lines and dots. *AUC* area under the curve, *RIMS2* Regulating Synaptic Membrane Exocytosis 2, *UCN2* Urocortin 2, *PGM5* Phosphoglucomutases, *CAVIN2* Caveolae Associated Protein 2, *CLIC6* Chloride Intracellular Channel 6, *GRIA1* Glutamate Ionotropic Receptor AMPA Type Subunit 1, *PKHD1L1* Polycystic Kidney and Hepatic Disease 1-Like 1

**Fig. 4:**
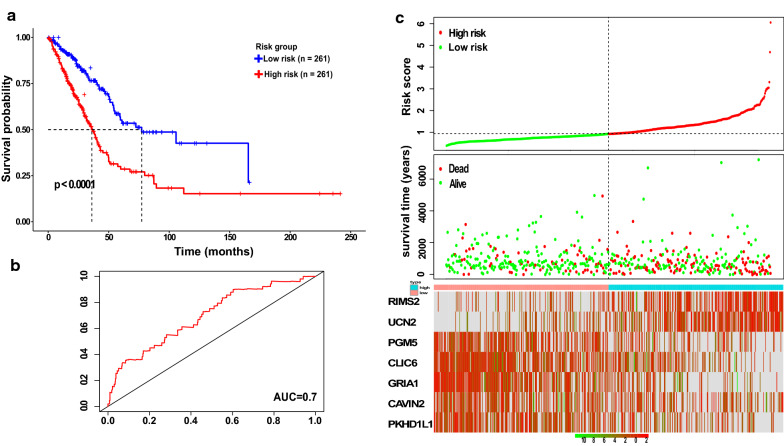
The prognostic performance of the 7-gene prognostic signature in the validation group. **a** Kaplan–Meier survival analysis of the seven-gene prognostic signature. **b** The receiver operating characteristic (ROC) curve analysis of seven-gene prognostic signature. **c** The distribution of risk scores (upper panel), the distribution of survival time (middle panel), and seven-gene expression profiles (bottom panel). Black dotted lines (median risk score) divide patients into low- and high-risk groups. Patients in the high-risk group are represented by red lines and dots. Patients in the low-risk group are represented by green lines and dots. *AUC* area under the curve, *RIMS2* Regulating Synaptic Membrane Exocytosis 2, *UCN2* Urocortin 2, *PGM5* Phosphoglucomutases, *CAVIN2* Caveolae Associated Protein 2, *CLIC6* Chloride Intracellular Channel 6, *GRIA1* Glutamate Ionotropic Receptor AMPA Type Subunit 1, *PKHD1L1* Polycystic Kidney and Hepatic Disease 1-Like 1

**Fig. 5:**
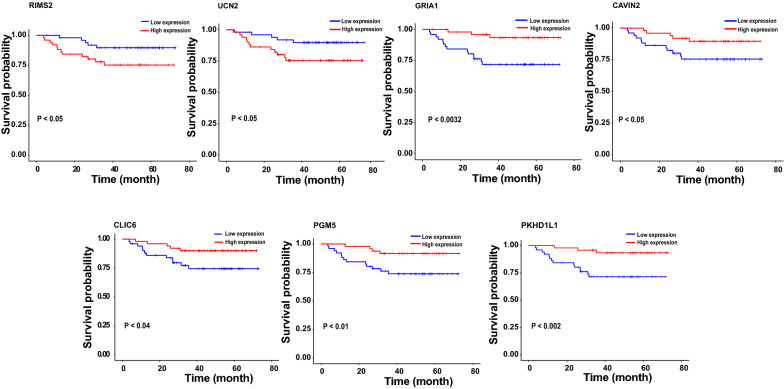
Kaplan–Meier survival analysis of the seven genes (RIMS2, UCN2, GRIA1, CAVIN2, CLIC6, PGM5, PKHD1L1). Long survival of LUAD patients was associated with low expression of RIMS2 and UCN2, while the overexpression of GRIA1, CAVIN2, CLIC6, PGM5, PKHD1L1 was associated with long survival of patients with LUAD

Based on the gene expression and regression coefficients of the seven genes from the multivariate cox analysis, we built a prognostic model to aid in the diagnosis of lung adenocarcinoma using the risk score approach. A risk score for each patient was given in the prognostic model. The median risk score of 0.7334 and 0.9367 were used as the cut-off points to classify the patients into high- and low-risk groups in the training (Fudan) (Fig. 3a) and validation (LUAD-TCGA) (Fig. 4a) groups, respectively. (Figs. 3c and 4c) show the distribution of the gene risk score, survival time, and the level of gene expression for seven genes in both training and validation groups respectively. Our findings revealed that there were significant differences in the OS status and gene expression levels for seven prognostic genes between the high and low-risk groups. In addition, the poor prognosis of LUAD is associated with the overexpression of RIMS2 and UCN2 (P = 0.05), and the low expression of each GRIA1, CAVIN2, CLIC6, PGM5, and PKHD1L1 (P < 0.05) (Fig. 5).

Patients who belong to the high-risk group had a significantly shorter OS than patients belonging to the low-risk group, as shown in Kaplan–Meier curves, with 29.4% higher risk and 3.9% lower risk of death for high- and low-risk groups, respectively (HR = 8.9456, 95% CI 2.041 to 39.2, P = 0.0004) (Table 4). The P value of one-side stratified log-rank test was 0.00037, confirming a significant difference between the high- and low-risk groups. Therefore, the clinical outcome of patients in the low-risk group was better than those in the high-risk group (Fig. 3a). The overall survival at 13 months was 98% (95% CI 94.2 to 1) and 84.3% (95% CI 74.9 to 94.9) in the low- and high-risk groups, respectively, and 68.6% (95% CI 56.4 to 83.5) in the high-risk group at 31 months (Table 4). For the TCGA validation group, Kaplan–Meier curves showed that overall survival was significantly longer in the low-risk group compared with the high-risk group, with 23.8% lower risk and 47.9% higher risk of death in the low- and high-risk groups, respectively (HR = 2.4139, 95% CI 1.779 to 3.276, P < 0.0001) (Table 4). The one-side stratified log-rank P value was < 0.0001, indicating the difference between the two groups (Fig. 4a). The median overall survival at 36 months was 49.9% (95% CI 42.95–58.1) for the high-risk group and 48.7% (95% CI 38.29–61.8) for the low-risk group at 77 months (Table 4). These findings suggest that the risk score of seven prognostic genes could be used as a prognostic marker. Furthermore, the time-dependent ROC curve was used to assess the predictive power of the seven prognostic genes for the overall survival prediction in training and validation groups. As shown in (Fig. 3b) the AUC for 5 years for overall survival in the training group was 0.91, and to confirm the predictive value of the gene signature, the TCGA-dataset group was used to test the finding, the result showed that the AUC for 5 years in the validation group was 0.7 (Fig. 4b). Thus, these results confirm that the 7 prognostic genes can be a prognostic predictor for LUAD.

**Table 4:** Overall survival, 7-gene signature, and Kaplan–Meier estimates

Variables	Groups	
Training group	Low risk (n = 51)	High risk (n = 51)
Deaths—no. (%)^a^	2	15
Data censored^b^	49	36
Median overall survival—mo (95% CI)	NE	NE
The overall survival (95% CI) by Kaplan–Meier estimation		
3.60 mo	NA	98 (94.3–1)
10.70 mo	NA	90.2 (82.4–98.7)
13 mo	98 (94.2–1)	84.3 (74.9–94.9)
23.57 mo	NA	82.3 (72.5–93.5)
27 mo	96 (90.7–1)	76.2 (65.3–88.9)
31 mo	NA	73.9 (62.6–87.2)
31.5 mo	NA	68.6 (56.4–83.5)

Validation group (TCGA data)	Low risk (n = 261)	High risk (n = 261)
Deaths—no. (%)^a^	62	125
Data censored^b^	199	136
Median overall survival—mo (95% CI)	77.3 (55.1–NE)	36 (31.6–42.2)
The overall survival (95% CI) by Kaplan–Meier estimation		
36–39.8 mo	75.4 (68.77–82.7)	49.9 (42.95–58.1)
77–79 mo	48.7 (38.29–61.8)	25.1 (17.72–35.6)
106–112 mo	42.6 (29.85–60.7)	15.2 (8.21–28.2)

NA indicate that there is no available events

NE represent that the value could not be estimated

^a^Represent the hazard ratio for death

^b^Indicate the date for censorship of patients on the date the patient was last known to be alive

### The signature of seven-genes as an independent predictive factor

**Fig. 6:**
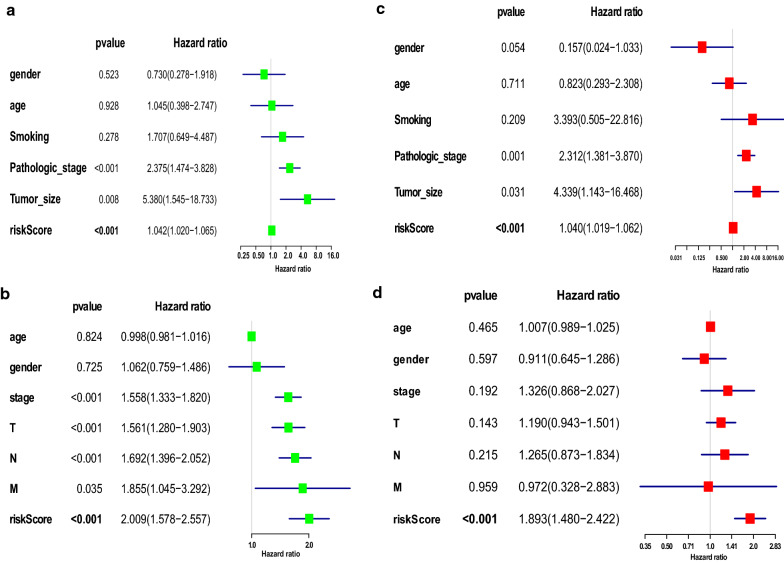
Cox regression analyses of risk score and clinical variables associated with the survival rate. **a**, **b** Univariate cox regression analyses in the training and validation groups, respectively. **c**, **d** Multivariate cox regression analyses in the training and validation groups, respectively

Univariate and multivariate cox regression analyses were implemented to evaluate the contribution of the seven-gene signature as an independent prognostic biomarker in the LUAD training group and LUAD TCGA validation group. The seven-gene signature and other clinicopathological factors, including sex, age, stage, tumor size, and smoking, were included as covariates in the training group. Sex, stage, age, stage T, stage N, and stage M were included as covariates in the validation group. Univariate regression analysis indicated that risk score, stage, and tumor size (risk score: P < 0.001, stage: P < 0.001, tumor size: p = 0.008, Fig. 6a) were significantly associated with patient survival in the LUAD training set. Risk score, T, N, M, and stage (risk score: P < 0.001, T: P < 0.001, N: P < 0.001, M: P = 0.035, and stage: P < 0.001, Fig. 6c) have significant correlation with OS of the LUAD-TCGA validation set. The corresponding multivariate cox regression analysis revealed and confirmed that pathological stage (HR = 2.312, 95% CI 1.381 − 3.870, P = 0.001, Fig. 6b), tumor size (HR = 4.339, 95% CI 1.143–16.468, P = 0.031, Fig. 6b), and risk score (HR = 1.040, 95% CI 1.019–1.062, P < 0.001, Fig. 6b) were significant independent risk factors of other clinical factors for the overall survival of the training group. Furthermore, multivariate cox regression analysis confirmed that only the risk score (HR = 1.893, 95% CI 1.480–2.422, P < 0.001, Fig. 6d) was an independent risk factor in the validation group. These results show the independence of the seven-gene signature as a risk factor for diagnosing patients with lung adenocarcinoma.

### Stratification analysis

**Fig. 7:**
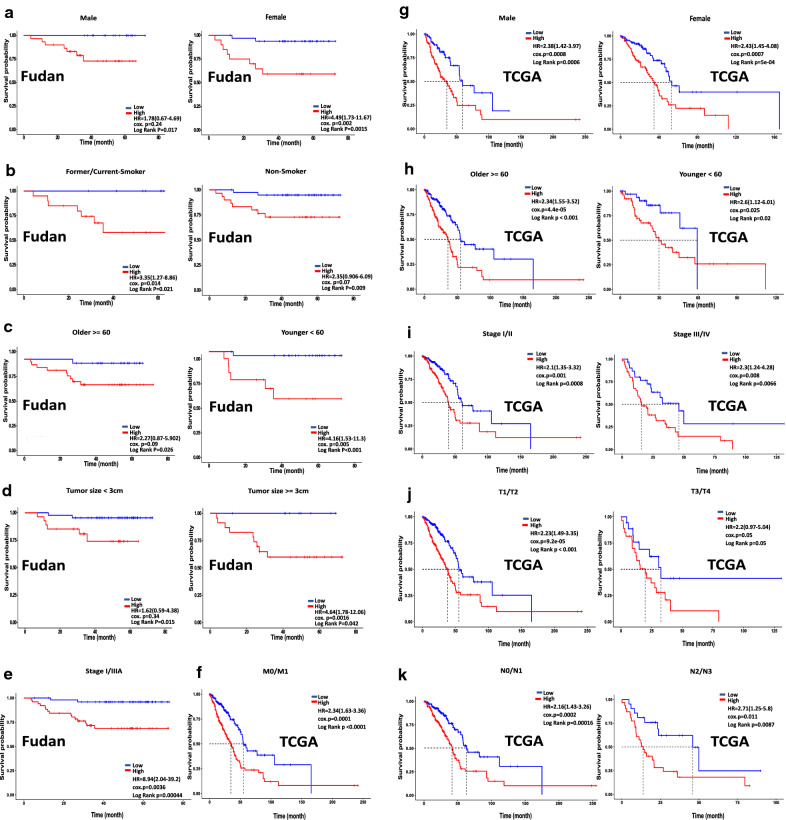
Kaplan–Meier survival analysis of the seven-gene risk score level for patients stratified by gender, history of smoking, age, tumor size and stage in the training group (Fudan). In the validation group (TCGA) the patients stratified by gender, age, stage, pT-stage, N-stage, M-stage

A stratification analysis was conducted to evaluate the ability of a seven-gene signature for predicting patient overall survival within the different subgroups. (Fig. 7) show that the seven-gene signature acts as a useful biomarker for predicting patient survival in the different subsets in the training group, non smokers [P = 0.009] and current/former-smokers [P = 0.021]; patients aged ≥ 60 years [P = 0.026] and age < 60 years [P < 0.001]; tumor size ≥ 3 cm [P = 0.042], tumor size < 3 cm [P = 0.015]; stage I/IIIA [P = 0.0004]; and male [P = 0.017] and female [P = 0.0015] and in the LUAD-TCGA group, age ≥ 60 [P < 0.001] and age < 60 [P < 0.02]; male [P = 0.0006] and female [P = 0.0005]; stage I/II [P = 0.00081] or stage III/IV [P = 0.0066]; T1/T2 [P < 0.001], T3/4 [P = 0.05], M0/M1 [P < 0.0001], N0/N1 [P = 0.00016], and N2/N3 [P = 0.0087]. The results showed that the seven-gene signature could stratify the patients in each subgroup into high- and low-risk groups. These results showed that patients in the high-risk group had a shorter and worse overall survival than those in the low-risk group. These results confirm the possibility of using this classification based on risk score to predict the overall survival of patients with LUAD.

### The correlation of prognostic seven genes with EGFR and KRAS mutations in LUAD patients

The results of mutation analysis (data not shown) using WES analysis for LUAD patients showed that 21 out of 102 patients had an EGFR mutation and two patients had a KRAS mutation. In order to investigate the relationship of gene expression of the seven genes, KRAS and EGFR mutations, we performed a combined analysis of gene expression and gene mutation. The results showed that the difference in the gene expression of the seven genes in the case of EGFR mutant and wild-type patients was observed only in the UCN2 P value = 0.049 (Additional file 3: Figure S1). Meanwhile,  additional file 4: Figure S2 showed a negative correlation between the gene expression of the seven genes and KRAS mutant and wild-type patients. This result indicates that EGFR and KRAS mutations have no impact on the gene expression and prognostic role of the seven prognostic genes.

### GO and signaling pathway enrichment analysis of 7-prognostic genes

GO functional enrichment analysis and KEEG pathway of the seven prognostic candidate genes were conducted by using online OmicsBean tool in order to identify the underlying GO terms process and pathways within these genes. The results showed that some genes were enriched in biological processes including regulation transport, regulation of localization, cyclic-nucleotide-mediated signaling and cAMP-mediated signaling (Additional file 5: Figure S3A), while some of the genes were enriched in molecular function including D4 dopamine receptor binding, AMPA glutamate receptor activity and G-protein coupled receptor binding (Additional file 5: Figure S3C). The main cell component in which some genes were enriched includes plasma membrane region, membrane region and cell junction (Additional file 5: Figure S3B). In addition, the result of KEGG analysis showed that different pathways were included but the main pathway was neuroactive ligand-receptor interaction (Additional file 5: Figure S3D).

## Discussion

When considering prognosis, NSCLC is believed to be an extremely heterogeneous disease where survival time among patients differs based on their pathological stages. Traditional clinicopathological variables, such as TNM level, tumor size, sex, age, as well as tumor factors, such as cell differentiation, vascular invasion, and vascularity, have been used in a broad framework to predict patient outcomes for diagnosis and treatment of patients with NSCLC. Predicting outcomes was insufficient due to the difference in effectiveness from different treatment strategies [29–31]. Consequently, inspecting molecular prognostic markers that reliably represent the biological traits of tumors is crucial for the treatment of patients with NSCLC, as well as for individualized prevention.

Previous studies have shown that molecular biomarkers and molecular signatures have received considerable interest from researchers and are used in clinical practice for many aspects of cancer, including tumorigenesis, progression, and prognosis [32]. Overall, almost all studies used the training group to develop and build the molecular signatures depend on the selection of overlapping genes in most databases, and this could lead to the recurrence of some genes in the new signatures; thus, this phenomenon may lead to similarity or convergence of the results, in addition to other concerns such as the absence of external independent verification, small sample size or effective verification that may hinder the efficiency and power of the prognostic model. In the current study, we established a 7-gene prognostic signature by selecting the genes that were significantly related to survival in patients with lung adenocarcinoma (LUAD) and have not been reported in the previous studies as prognostic genes to predict overall survival in LUAD patients. The consistent finding was achieved in another independent group of LUAD patients from the TCGA database. Our seven-gene prognostic signature significantly identified the high and low-risk LUAD patients with significant differences in overall survival. The ROC curve showed that the predictive performance of the 7-gene prognostic signature as a prognostic marker was superior both in Fudan and TCGA datasets, these results indicate compatibility in our signature between both data. Stratification analysis and cox regression (univariate and multivariate) analysis showed that the 7-gene prognostic signature was an independent prognostic marker. Our results suggested that a gene signature based on seven genes can be sufficiently effective and promising prognostic biomarker of survival in lung adenocarcinoma patients.

Commonly, prognostic gene signatures [33, 34] classify patients into high or low-risk groups. Zuo et al. [20] identified a six-gene signature; however, the AUC was 0.749, 0.685, and 0.667 in the three independent datasets GSE31210, GSE37745, and GSE50081, respectively. Li et al. [35] identified an eight-gene prognostic signature that may act as prognostic marker of patients with lung adenocarcinoma. Xie et al. [18] identified a six-gene signature based on integrated analysis and weight gene co-expression network. The AUC was 0.99 and 0.82 or 0.77 and 0.75 in predicting 1–10 years of survival of TCGA-LUAD and GSE11969 datasets, respectively. Jiang et al. [36] identified a gene signature of 10 genes, where this 10-gene signature was able to classify patients into a high-risk group and a low-risk group. The predictive power of this signature were 0.753, 0.724, and 0.73 on the basis of AUC for 1, 3 and 5 years survival respectively. Zhang et al. [37] identified a gene signature of nine genes that helps predict poor prognosis for lung adenocarcinoma patients. The AUC was 0.71. Liu et al. [38] established a four-gene signature related to glycolysis that can predict the outcome of patients with lung adenocarcinoma. Li et al. [39] established an eight-miRNA signature to predict survival for LUAD patients where the AUC for 5 years was 0.626, however, this signature lacked the external validation in an independent group. Peng et al. [40] developed a robust prognostic signature consisting of two lncRNAs (C1orf132 and TMPO-AS1) for stage I–II LUAD patients without receiving adjuvant therapy. By contrast, the AUC of our seven-gene signature was higher given that seven genes were used, which makes it suitable for clinical application.

The seven genes in our signature consist of UCN2 and RIMS2 as risk factors and CAVIN2, GRIA1, PKHD1L1, PGM5, and CLIC6 as protective factors. CLIC6 is a member of the intracellular chloride channels consisting one of the dopamine receptor-mediated signaling pathways and has changed its expression in breast cancer [41, 42]. The prognosis of patients’ cancer outcomes has not been reported previously. Chen Zheng et al. [43] reported that PKHD1L1 may be a PTC-associated tumor suppressor gene and a potential molecular biomarker useful as a therapeutic target in the coming years. PGM5 is a diagnostic and prognostic biomarker independently associated with the survival of patients with liver cancer [44] and colorectal cancer [45]. Tilley et al. [46] reported that increased expression and hypermethylation of GRIA1 was correlated with survival in patients with basal-like bladder cancer and was used as a prognostic biomarker. Another report for Yang et al. [47] showed that GRIA1 is one of the top 10 target genes in the protein–protein interaction network present in the five-miRNA signature model used as a novel prognosis biomarker and therapeutic target for patients with colorectal cancer. Codenotti et al. [48] reported that CAVIN2 is a useful marker for discriminating the degree of differentiation in liposarcoma tumors. Annabi et al. [49] highlighted the role of CAVIN2 in the regulation of each inflammatory and angiogenic for TNF-activated MSC. No previous reports are related to the prognosis of cancer outcomes in patients. Esnault et al. [50] reported that UCN2 has the downstream function of inflammation, tissue remodeling, and lipid synthesis in human lung fibroblasts. On the other hand, our result of the UCN2 did not compatible to the previous study of Hao et al. [51] and this may be attributed to the different study conditions, more verification in the future is needed to confirm the results. No previous survival prediction studies have been reported for patients with cancer. RIMS2 has been reported to be mutated in melanoma [52], and no other studies on the prediction of outcomes in patients with cancer have been reported.

We further explored the correlation among patients with KRAS, EGFR mutations and the predictive value of the seven genes. The results showed a negative correlation between the predictive value of the seven genes and KRAS, while only the UCN2 predictive value showed a positive correlation with EGFR. These results suggested that the predictive values of the seven genes are independent and there is no effect of both mutations on the gene expression of these genes as well as their prediction role. Subsequent GO and KEGG enrichment analysis indicate that genes in the prognostic model were enriched in the different biological functions including regulation, cyclic-nucleotide-mediated signaling, cAMP-mediated signaling, cell junction, plasma membrane region and membrane region, D4 dopamine receptor binding, AMPA glutamate receptor activity and G-protein coupled receptor binding and neuroactive ligand-receptor interaction pathway. These enrichment findings indicated that the oncogenesis and development of LUAD may be mediated by these biological functions. However, the mechanism that binds genes to each other is still unknown and needs further research in the future.

Overall, our study has established an accurate and effective 7-gene prognostic signature to predict survival for LUAD patients by using genes related to survival that are not reported in previous studies. The risk score based on these seven prognostic genes is characterized by a good predictive performance and it was able to effectively distinguish high-risk LUAD patients from low-risk patients in addition to its ability to stratify patients in the subgroups making it a useful tool for follow-up monitoring and prognosis of LUAD patients and reducing the excessive cost of molecular diagnosis. In addition, the seven genes and their participation in the prognosis of the LUAD and predicting the patients survival have not been reported in the literature, therefore, our study is the first to identify the predictability of the seven genes and their independence from the other clinical features in the prediction. However, like any other research work, there are some limitations to our study; first, since our study relied mainly on computational analysis, it is necessary to achieve these results through further biological experiments in the future; second, the potential biological mechanisms and pathways linking the seven genes in the prognostic signature are still unclear and need further investigation.

## Conclusions

In summary, we proposed a new 7-gene prognostic signature as an independent prognostic biomarker characterized by good predictive performance to predict the overall survival of LUAD patients. The 7-gene prognostic signature may help with early detection, accurately assess patient diagnosis, contribute to follow-up monitoring and help clinicians make effective decisions regarding the potential individual treatment of LUAD patients, which improves their survival. In addition, these genes may be used as therapeutic targets in the future.

## Supplementary Information

**Additional file 1: Table S1:** Source data underlying all figures in the analysis.

**Additional file 2: Table S2:** The 31 unreported prognostic genes associated with lung adenocarcinoma patients survival.

**Additional file 3: Figure S1.** The relationship between the gene expression of the seven genes in the prognostic model and the EGFR mutation in LUAD patients.

**Additional file 4: Figure S2.** The relationship between the gene expression of the seven genes in the prognostic model and the KRAS mutation in LUAD patients.

**Additional file 5: Figure S3.** Functional enrichment analysis of the seven prognostic genes associated with overall survival in LUAD patients. (A) Biological process, (B) Cell component, (C) Molecular function, (D) KEGG pathway enrichment analysis. Dotplot indicates the counts of genes.

## Data Availability

The raw data used and/or analysed during the current study could be obtained from the European Genome-phenome Archive (EGA) with the accession code EGAS00001004006. The LUAD-TCGA dataset used in this study could be obtained from TCGA Database (https://portal.gdc.cancer.gov/). Source data underlying all figures are provided as an additional file 1: Table S1.

## Abbreviations

AUC: Area under curve
bTMB: Blood tumor mutation burden
CAVIN2: Caveolae associated protein 2
CLIC6: Chloride intracellular channel 6
CI: Confidence interval
CRC: Colorectal cancer
DEGs: Differentially expressed genes
FDR: False discovery rate
GRIA1: Glutamate ionotropic receptor AMPA type subunit 1
HLFs: Human lung fibroblasts
HR: Hazard ratio
K-M: Kaplan–Meier
LPS: Liposarcoma
LUAD: Lung adenocarcinoma
LUSC: Lung squamous cell carcinoma
NSCLC: Non-small lung cancer
OS: Overall survival
PGM5: Phosphoglucomutases
PKHD1L1: Polycystic kidney and hepatic disease 1-like 1
RIMS2: Regulating synaptic membrane exocytosis 2
SCLC: Small cell lung cancer
TCGA: The Cancer Genome Atlas
TMB: Tumor mutation burden
UCN2: Urocortin 2

## References

[1] Torre LA, Bray F, Siegel RL, Ferlay J, Lortet-Tieulent J, Jemal A (2015). Global cancer statistics, 2012. CA Cancer J Clin.

[2] Herbst RS, Morgensztern D, Boshoff C (2018). The biology and management of non-small cell lung cancer. Nature.

[3] Hou S, Zhou S, Qin Z, Yang L, Han X, Yao S, Ji H (2017). Evidence, mechanism, and clinical relevance of the transdifferentiation from lung adenocarcinoma to squamous cell carcinoma. Am J Pathol.

[4] Nicoleau S, Wojciak-Stothard B (2020). Beyond thrombosis: the role of platelets in pulmonary hypertension. Sci Med J.

[5] Pullamsetti SS, Kojonazarov B, Storn S, Gall H, Salazar Y, Wolf J (2017). Lung cancer-associated pulmonary hypertension: role of microenvironmental inflammation based on tumor cell-immune cell cross-talk. Sci Transl Med.

[6] Bray F, Ferlay J, Soerjomataram I, Siegel RL, Torre LA, Jemal A (2018). Global cancer statistics 2018: GLOBOCAN estimates of incidence and mortality worldwide for 36 cancers in 185 countries. CA Cancer J Clin.

[7] Lin HT, Liu FC, Wu CY, Kuo CF, Lan WC, Yu HP (2019). Epidemiology and survival outcomes of lung cancer: a population-based study. Biomed Res Int.

[8] Siegel RL, Miller KD, Jemal A (2019). Cancer statistics, 2019. CA Cancer J Clin.

[9] Siegel RL, Miller KD, Fuchs HE, Jemal A (2021). Cancer statistics, 2021. CA Cancer J Clin.

[10] Travis WD, Brambilla E, Nicholson AG, Yatabe Y, Austin JHM, Beasley MB (2015). The 2015 World Health Organization classification of lung tumors: impact of genetic, clinical and radiologic advances since the 2004 classification. J Thorac Oncol.

[11] Gandhi L, Rodríguez-Abreu D, Gadgeel S (2018). Pembrolizumab plus chemotherapy in metastatic non-small-cell lung cancer. N Engl J Med.

[12] Yoshizawa A, Motoi N, Riely GJ, Sima CS, Gerald WL, Kris MG (2011). Impact of proposed IASLC/ATS/ERS classification of lung adenocarcinoma: prognostic subgroups and implications for further revision of staging based on analysis of 514 stage I cases. Mod Pathol.

[13] Zhang WC, Shyh-Chang N, Yang H, Rai A, Umashankar S, Ma S (2012). Glycine decarboxylase activity drives non-small cell lung cancer tumor-initiating cells and tumorigenesis. Cell.

[14] Chen R, Khatri P, Mazur PK, Polin M, Zheng Y, Vaka D (2014). A meta-analysis of lung cancer gene expression identifies PTK7 as a survival gene in lung adenocarcinoma. Cancer Res.

[15] Liu GM, Zeng HD, Zhang CY, Xu JW (2019). Identification of a six-gene signature predicting overall survival for hepatocellular carcinoma. Cancer Cell Int.

[16] Gettman MT, Blute ML, Spotts B, Bryant SC, Zincke H (2001). Pathologic staging of renal cell carcinoma: significance of tumor classification with the 1997 TNM staging system. Cancer.

[17] Soria JC, Ohe Y, Vansteenkiste J, Reungwetwattana T, Chewaskulyong B, Lee KH (2018). Osimertinib in untreated EGFR-mutated advanced non-small-cell lung cancer. N Engl J Med.

[18] Xie H, Xie C (2019). A six-gene signature predicts survival of adenocarcinoma type of non-small-cell lung cancer patients: a comprehensive study based on integrated analysis and weighted gene coexpression network. Biomed Res Int.

[19] Sun R, Meng X, Wang W, Liu B, Lv X, Yuan J (2019). Five genes may predict metastasis in non-small cell lung cancer using bioinformatics analysis. Oncol Lett.

[20] Zuo S, Wei M, Zhang H, Chen A, Wu J, Wei J, Dong J (2019). A robust six-gene prognostic signature for prediction of both disease-free and overall survival in non-small cell lung cancer. J Transl Med.

[21] Chen H, Carrot-Zhang J, Zhao Y (2019). Genomic and immune profiling of pre-invasive lung adenocarcinoma. Nat Commun.

[22] Dobin A, Davis CA, Schlesinger F (2013). STAR: ultrafast universal RNA-seq aligner. Bioinformatics.

[23] Liao Y, Smyth GK, Shi W (2014). featureCounts: an efficient general purpose program for assigning sequence reads to genomic features. Bioinformatics.

[24] Alì G, Bruno R, Poma AM (2019). Whole transcriptome targeted gene quantification provides new insights on pulmonary sarcomatoid carcinomas. Sci Rep.

[25] Győrffy B, Surowiak P, Budczies J, Lánczky A (2013). Online survival analysis software to assess the prognostic value of biomarkers using transcriptomic data in non-small-cell lung cancer. PLoS ONE.

[26] Cao Y, Zhu W, Chen W (2019). Prognostic value of BIRC5 in lung adenocarcinoma lacking EGFR, KRAS, and ALK mutations by integrated bioinformatics analysis. Dis Markers.

[27] Wang L, Qu J, Liang Y (2020). Identification and validation of key genes with prognostic value in non-small-cell lung cancer via integrated bioinformatics analysis. Thorac Cancer.

[28] Guo JC, Wu Y, Chen Y (2018). Protein-coding genes combined with long noncoding RNA as a novel transcriptome molecular staging model to predict the survival of patients with esophageal squamous cell carcinoma. Cancer Commun (Lond).

[29] Rami-Porta R, Bolejack V, Crowley J (2015). The IASLC lung cancer staging project: proposals for the revisions of the T descriptors in the forthcoming eighth edition of the TNM classification for lung cancer. J Thorac Oncol.

[30] Tas F, Ciftci R, Kilic L, Karabulut S (2013). Age is a prognostic factor affecting survival in lung cancer patients. Oncol Lett.

[31] Radkiewicz C, Dickman PW, Johansson ALV (2019). Sex and survival in non-small cell lung cancer: a nationwide cohort study. PLoS ONE.

[32] Zhu CQ, Tsao MS (2014). Prognostic markers in lung cancer: is it ready for prime time?. Transl Lung Cancer Res.

[33] Wang J, Chen X, Tian Y (2020). Six-gene signature for predicting survival in patients with head and neck squamous cell carcinoma. Aging (Albany NY).

[34] Zhang Z, Lin E, Zhuang H (2020). Construction of a novel gene-based model for prognosis prediction of clear cell renal cell carcinoma. Cancer Cell Int.

[35] Li S, Xuan Y, Gao B, Sun X, Miao S, Lu T, Wang Y, Jiao W (2018). Identification of an eight-gene prognostic signature for lung adenocarcinoma. Cancer Manag Res.

[36] Jiang H, Xu S, Chen C (2020). A ten-gene signature-based risk assessment model predicts the prognosis of lung adenocarcinoma. BMC Cancer.

[37] Zhang L, Zhang Z, Yu Z (2019). Identification of a novel glycolysis-related gene signature for predicting metastasis and survival in patients with lung adenocarcinoma. J Transl Med.

[38] Liu C, Li Y, Wei M, Zhao L, Yu Y, Li G (2019). Identification of a novel glycolysis-related gene signature that can predict the survival of patients with lung adenocarcinoma. Cell Cycle.

[39] Li X, Shi Y, Yin Z, Xue X, Zhou B (2014). An eight-miRNA signature as a potential biomarker for predicting survival in lung adenocarcinoma. J Transl Med.

[40] Peng F, Wang R, Zhang Y, Zhao Z, Zhou W, Chang Z, Liang H, Zhao W, Qi L, Guo Z, Gu Y (2017). Differential expression analysis at the individual level reveals a lncRNA prognostic signature for lung adenocarcinoma. Mol Cancer.

[41] Low SK, Chin YM, Ito H, Matsuo K, Tanikawa C, Matsuda K (2019). Identification of two novel breast cancer loci through large-scale genome-wide association study in the Japanese population. Sci Rep.

[42] Ko JH, Ko EA, Gu W, Lim I, Bang H, Zhou T (2013). Expression profiling of ion channel genes predicts clinical outcome in breast cancer. Mol Cancer.

[43] Zheng C, Quan R, Xia EJ, Bhandari A, Zhang X (2019). Original tumour suppressor gene polycystic kidney and hepatic disease 1-like 1 is associated with thyroid cancer cell progression. Oncol Lett.

[44] Jiao Y, Li Y, Jiang P, Han W, Liu Y (2019). PGM5: a novel diagnostic and prognostic biomarker for liver cancer. PeerJ.

[45] Sun Y, Long H, Sun L, Sun X, Pang L, Chen J, Yi Q, Liang T, Shen Y (2019). PGM5 is a promising biomarker and may predict the prognosis of colorectal cancer patients. Cancer Cell Int.

[46] Tilley SK, Kim WY, Fry RC (2017). Analysis of bladder cancer tumor CpG methylation and gene expression within The Cancer Genome Atlas identifies GRIA1 as a prognostic biomarker for basal-like bladder cancer. Am J Cancer Res.

[47] Yang G, Zhang Y, Yang J (2019). A Five-microRNA signature as prognostic biomarker in colorectal cancer by bioinformatics analysis. Front Oncol.

[48] Codenotti S, Vezzoli M, Poliani PL, Cominelli M, Monti E, Fanzani A (2017). Cavin-2 is a specific marker for detection of well-differentiated liposarcoma. Biochem Biophys Res Commun.

[49] Annabi B, Zgheib A, Annabi B (2017). Cavin-2 functions as a suppressive regulator in TNF-induced mesenchymal stromal cell inflammation and angiogenic phenotypes. Int J Stem Cells.

[50] Esnault S, Bernau K, Torr EE, Bochkov YA, Jarjour NN, Sandbo N (2017). RNA-sequencing analysis of lung primary fibroblast response to eosinophil-degranulation products predicts downstream effects on inflammation, tissue remodeling and lipid metabolism. Respir Res.

[51] Hao Z, Huang Y, Cleman J (2008). Urocortin2 inhibits tumor growth via effects on vascularization and cell proliferation. Proc Natl Acad Sci U S A.

[52] Zhang D, Xia J (2020). Somatic synonymous mutations in regulatory elements contribute to the genetic aetiology of melanoma. BMC Med Genomics.

