# Supplementary figures and images for: A seven-gene prognostic signature predicts overall survival of patients with lung adenocarcinoma (LUAD)
Source: Cancer Cell Int. 2021 Jun 6;21:294. doi: 10.1186/s12935-021-01975-z (PMC8183047; doi:10.1186/s12935-021-01975-z)

Wilcoxon,  $P = 0.29$ 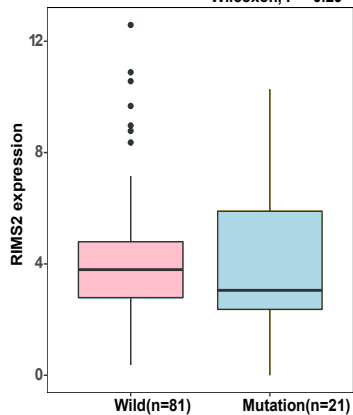Wilcoxon,  $P = 0.049$ 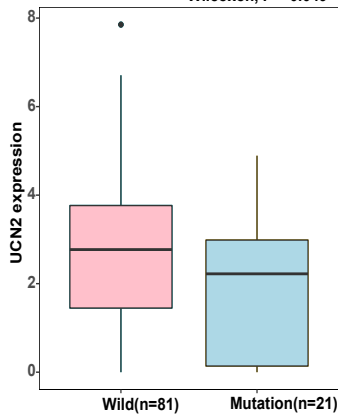Wilcoxon,  $P = 0.07$ 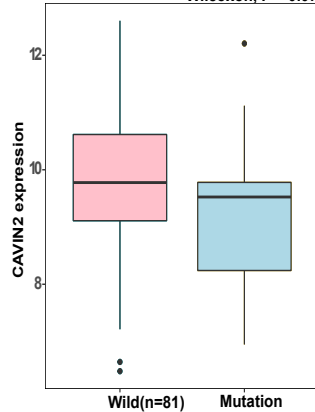Wilcoxon,  $P = 0.2$ 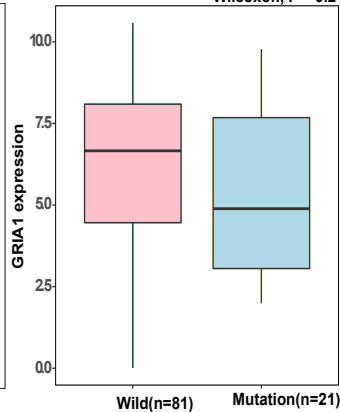Wilcoxon,  $P = 0.43$ 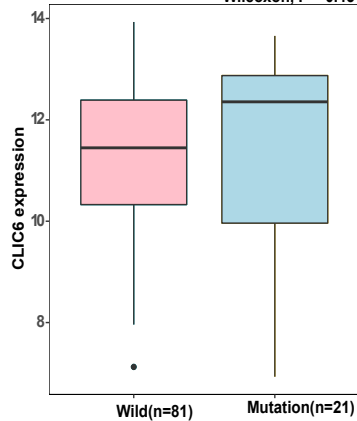Wilcoxon,  $P = 0.075$ 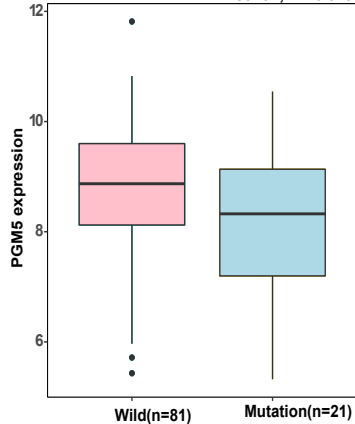Wilcoxon,  $P = 0.066$ 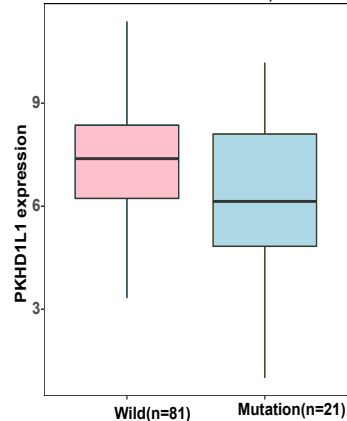

Supplement: Supplementary file 3 — Additional file 3: Figure S1. The relationship between the gene expression of the seven genes in the prognostic model and the EGFR mutation in LUAD patients. [file 12935_2021_1975_MOESM3_ESM.pdf]

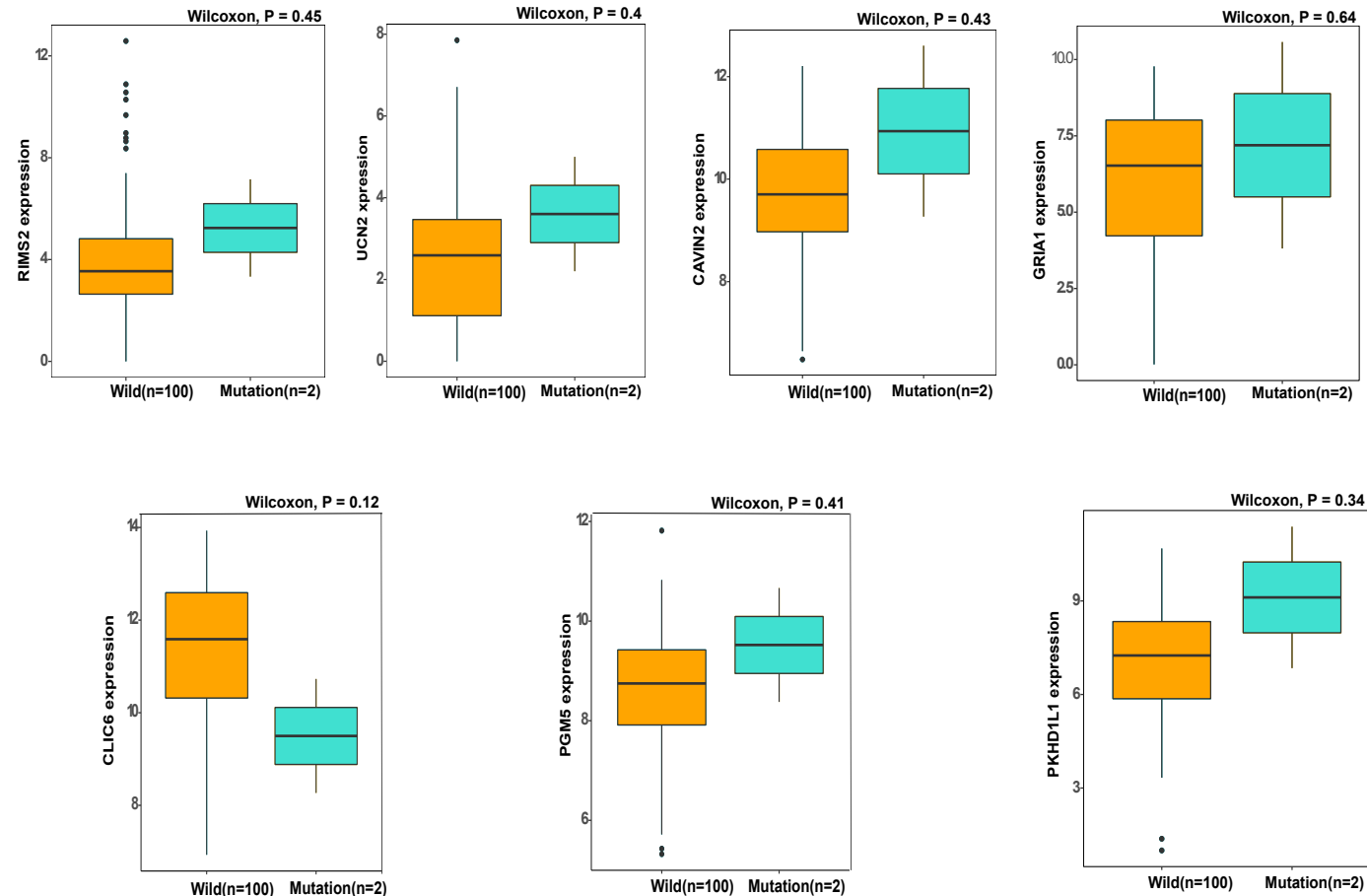

Supplement: Supplementary file 4 — Additional file 4: Figure S2. The relationship between the gene expression of the seven genes in the prognostic model and the KRAS mutation in LUAD patients. [file 12935_2021_1975_MOESM4_ESM.pdf]

A)

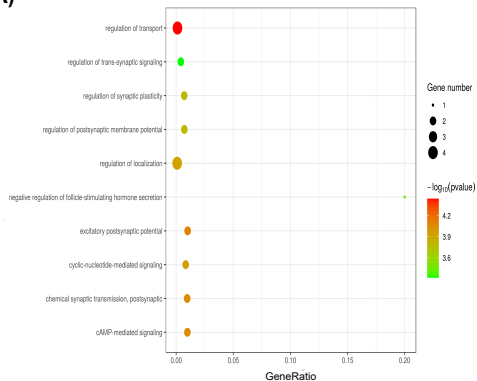

C)

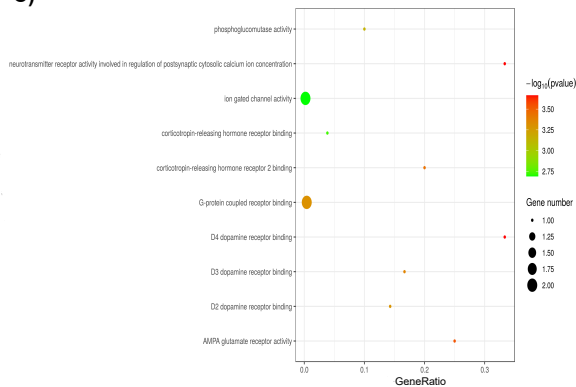

B)

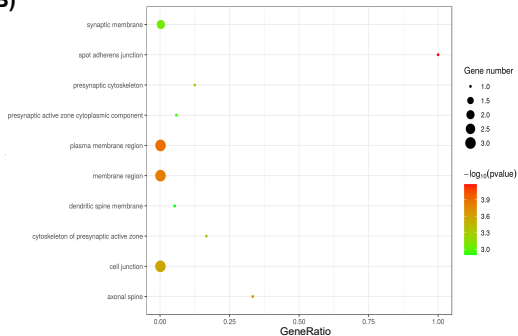

D)

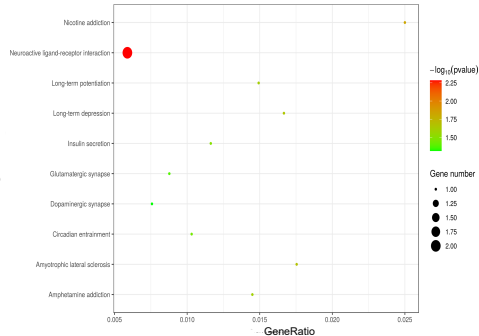

Supplement: Supplementary file 5 — Additional file 5: Figure S3. Functional enrichment analysis of the seven prognostic genes associated with overall survival in LUAD patients. (A) Biological process, (B) Cell component, (C) Molecular function, (D) KEGG pathway enrichment analysis. Dotplot indicates the counts of genes. [file 12935_2021_1975_MOESM5_ESM.pdf]
